# LDL-C: lower is better for longer—even at low risk

**DOI:** 10.1186/s12916-020-01792-7

**Published:** 2020-10-08

**Authors:** Peter E. Penson, Matteo Pirro, Maciej Banach

**Affiliations:** 1 School of Pharmacy and Biomolecular Sciences, Liverpool John Moores University, Liverpool, UK; 2 Liverpool Centre For Cardiovascular Science, Liverpool, UK; 3 University of Liverpool, Liverpool, UK; 4 Unit of Internal Medicine, Angiology and Arteriosclerosis Diseases, Department of Medicine, University of Perugia, Perugia, Italy; 5 Department of Hypertension, Medical University of Lodz, Rzgowska 281/289, 93-338 Lodz, Poland; 6 Polish Mother’s Memorial Hospital Research Institute (PMMHRI), Lodz, Poland; 7 Cardiovascular Research Centre, University of Zielona Gora, Zielona Gora, Poland

**Keywords:** Cardiovascular disease, Ezetimibe, Low risk, Risk stratification, Statins

## Abstract

**Background:**

Low-density lipoprotein cholesterol (LDL-C) causes atherosclerotic disease, as demonstrated in experimental and epidemiological cohorts, randomised controlled trials, and Mendelian randomisation studies.

**Main text:**

There is considerable inconsistency between existing guidelines as to how to effectively manage patients at low overall risk of cardiovascular disease (CVD) who have persistently elevated levels of LDL-C.

We propose a step-by-step practical approach for the management of cardiovascular risks in individuals with low (< 1%) 10-year risk of CVD, and elevated (> 140 mg/dL, 3.6 mmol/L) LDL-C. The strategy proposed is based on the level of adherence to lifestyle interventions (LSI), and in case of non-adherence, stepwise practical management, including lipid-lowering therapy, is recommended to achieve a target LDL-C levels (< 115 mg/dL, 3.0 mmol/L).

**Conclusions:**

Further studies are necessary to answer the questions on the long-term efficacy, safety, and cost-effectiveness of the suggested approach. This is critical, considering the ever-increasing numbers of such low-risk patients seen in clinical practice.

## Background

Low-density lipoprotein cholesterol (LDL-C) causes atherosclerotic disease. This fact has been repeatedly demonstrated in experimental studies, epidemiological cohorts, randomised clinical trials of LDL-C lowering drugs, and studies employing Mendelian randomisation [1]. Notably, the gradient of the relationship between LDL-C and outcomes of atherosclerotic disease becomes steeper with increasing duration of follow-up (in epidemiological studies) and treatment (in intervention trials). From this, it can be concluded that an individual’s risk of atherosclerotic disease is strongly determined by their cumulative lifelong exposure to LDL-C [1]. Accordingly, a significant long-term increased risk for coronary heart disease (CHD) and cardiovascular mortality has been reported in young adults with LDL-C ≥ 100 mg/dL (2.5 mmol/L) [2, 3]. Therefore, in order to prevent atherosclerosis and its sequelae (myocardial infarction, ischemic stroke and peripheral arterial disease), it is necessary to act early in life. Indeed, the early manifestations of atherosclerosis are often apparent in the third decade of life [4–7], a problem that is brought into stark reality by the early morbidity and mortality associated with familial hypercholesterolaemia (FH) [8]. Moreover, changes in plasma cholesterol levels have been found to be directly associated with cardiovascular disease (CVD) events in young adults [9]. Based on these findings, it is clear that, with respect to LDL-C, ‘lower is better, for longer’.

## Main text

The risk of atherosclerotic CVD events and mortality can be reduced by currently used drugs, which reduce circulating concentrations of LDC. These include statins (which inhibit HMG-CoA reductase, the rate-limiting step in hepatic endogenous cholesterol synthesis) [10–12], ezetimibe (which inhibits the polytopic transmembrane protein, Niemann-Pick C1-Like 1, which is responsible for cholesterol absorption from the jejunal brush border) [13], and anti-proprotein convertase subtilisin/kexin type 9 (PCSK9) monoclonal antibodies (which inhibit PCSK9, a regulatory protein that binds to LDL-receptors on hepatocytes and promotes their routing into lysosomes for proteolytic destruction) [14, 15]. Intriguingly, inhibition of HMG-CoA reductase, Niemann-Pick C1-Like 1 and PCSK9 all increase the LDL-receptor density on the surface of hepatocytes, which results in more extensive removal of circulating LDL particles.

Because of the multifactorial nature of risk factors predisposing to CVD, and in order to use lipid-lowering drugs most effectively, reducing unnecessary cost and exposure to adverse effects, national and international guidelines make recommendations relating to the initiation of lipid-lowering therapy based upon calculations of cardiovascular risk, rather than lipid profile alone. Although the specifics of the risk prediction tools and thresholds differ between major guidelines, the approach is essentially the same. The European Society of Cardiology (ESC)/European Atherosclerosis Society (EAS) [16] recommendations are based upon the SCORE 10-year risk calculation [17] and consider both the overall SCORE and the untreated circulating LDL-C concentration in determining whether drug interventions should be employed [16]. In the UK, NICE recommends offering lipid modification therapy to people aged 84 years and younger if their estimated 10-year risk of developing CVD using the QRISK [18] assessment tool is 10% or more and lifestyle modification is ineffective or inappropriate [19]. American College of Cardiology (ACC)/American Heart Association (AHA) guidelines recommend statin therapy when 10-year risk, calculated using the pooled-cohort equations [20] exceeds 7.5% [21] (for non-diabetics with LDL-C > 70 mg/dL [1.8 mmol/L] and the presence of other risk enhancing factors).

However, 10-year predictions underestimate lifetime risk and are driven to a large extent by age [22, 23]. Clinical guidelines based upon 10-year risk profiles are prone to ignore younger patients with a single risk factor, which is unlikely to result in deleterious outcomes in the forthcoming decade, but which epidemiological evidence suggests is associated with an elevated incidence of disease later in life [24]. This is perhaps justifiable for non-modifiable risk factors, but when multiple strategies exist to ameliorate the associated risk, as they do for LDL-C, then we are at risk of missing opportunities to intervene now to improve health in later life.

There is considerable inconsistency in approaches used to manage patients at overall low risk of CVD (< 1%) who have persistently elevated levels of LDL-C (115 mg/dL [3 mmol/L]–190 mg/dL [4.9 mmol/L]). In the 2016 ESC/EAS guidelines for the management of dyslipidaemias, no intervention was recommended until LDL-C reached 190 mg/dL (4.9 mmol/L) [25], which was a matter of considerable debate and presented problems in everyday clinical practice. In the 2019 recommendations, lifestyle modifications are suggested initially, and pharmacological interventions can be now considered for individuals with LDL-C above 115 mg/dL (3 mmol/L) [16].

**Fig. 1:**
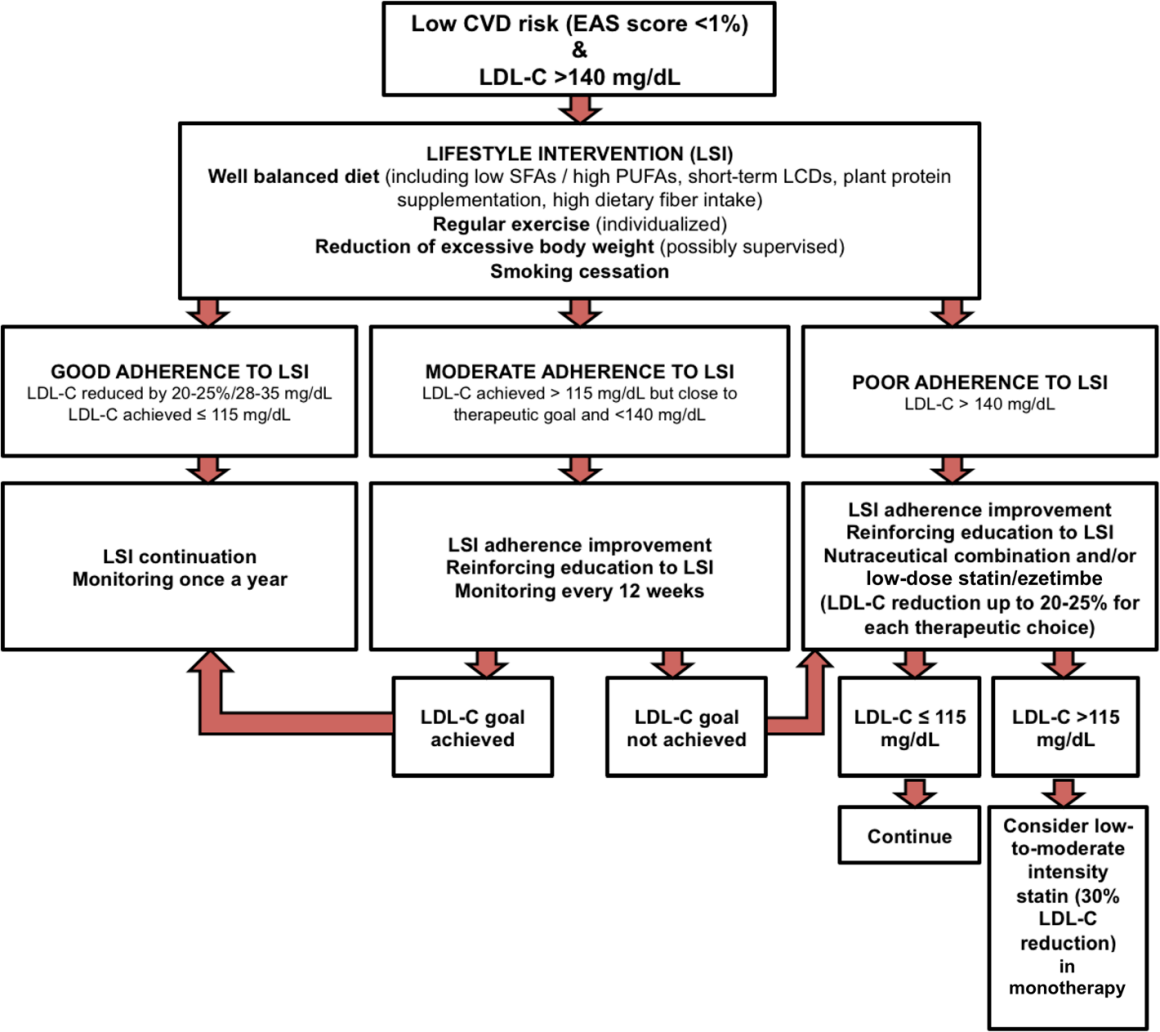
Proposed approach to the management of cardiovascular risks in individuals with low (< 1%) 10-year risk of cardiovascular disease, but elevated (> 140 mg/dL, 3.6 mmol/L) LDL-C

We propose a more detailed approach to the management of cardiovascular risks in individuals with low (< 1%) 10-year risk of CVD, but elevated (> 140 mg/dL, 3.6 mmol/L) LDL-C (Fig. 1). The cutoff point of 140 mg/dL (3.6 mmol/L) results from the calculations of the possibility of LDL-C reduction to the target level of 115 mg/dL (3 mmol/L) with the adherent lifestyle changes; this level also appeared in the previous 2017 ESC/EAS task force on practical clinical guidance for PCSK9 inhibitors application in patients with atherosclerotic CVD, where the persistent LDL-C level of > 140 mg/dL (3.6 mmol/L; on statins and ezetimibe) was an independent risk factor linked to significantly increased CVD risk, requiring consideration of PCSK9 inhibitors [26]. The strategy proposed in this paper is largely based upon evidence-based interventions to reduce LDL-C, rather than the ideal endpoints of major adverse cardiovascular events (MACE) and mortality. In this regard, the strong-graded relationship between LDL-C and mortality seen across many hundreds of studies [1], and the availability of very large well-conducted epidemiological datasets means that it is appropriate to base recommendations on studies employing LDL-C reduction as the primary endpoint. The potential benefits of this approach are highlighted by a recent study which quantified the relative importance of risk factors for CHD. Amongst a population of 22,626 individuals, aged 45–85, the population attributable fraction (PAF) was found to be 17% (95% CI 10.2–23.2) for non-HDL-C > 130 mg/dl. However, in the subpopulation of individuals aged 45–54, the PAF was 32.8% (95% CI 10.1–49.9), suggesting a large proportion of coronary heart disease might be eliminated by optimal lipid-management in younger individuals [27].

The first and most important approach to low 10-year risk individuals is to provide education and information relating to the benefits of lifestyle interventions (LSI). The dietary approach to LDL-C reduction includes the promotion of a well-balanced diet, which derives a low proportion of energy from saturated fatty acids [28], and a high proportion from polyunsaturated fatty acids [29]. ESC/EAS guidelines suggest that carbohydrate intake should range between 45 and 55% of total energy intake and that intake of added sugar should not exceed 10% of total energy [16]. In the short-term, low-carbohydrate diets (LCD) [30] improve lipid profile, although a recent well-designed investigation found a potentially unfavourable association of LCD with overall and cause-specific mortality, based on both analyses of an established cohort and by pooling previous cohort studies in a meta-analysis [31, 32]. Dietary fibre (particularly when it replaces saturated fat in the diet) has beneficial effects on plasma lipid profile [33]. The benefits of smoking cessation [34], exercise [35], and weight reduction [35], on cardiovascular risk, are more closely associated with elevation of HDL-C and reduction of triglycerides, than reduction of LDL-C, but these strategies should nevertheless be encouraged in all individuals, especially those with any risk factors for CVD [16, 25].

For those individuals who manage good adherence to LSI (indicated by a reduction of LDL-C by 20–25%/28–35 mg/dl = achieved LDL-C ≤ 115 mg/dL [3 mmol/L]), annual check-up to monitor cardiovascular risk and reinforce messages about LSI are appropriate. For individuals with moderate success at reducing LDL-C using LSI (indicated by LDL-C > 115 mg/dL [3 mmol/L] but close to target and < 140 mg/dL [3.6 mmol/L]), more frequent monitoring (preferably every 12 weeks) is appropriate, and these individuals may benefit from enhanced educational interventions. Where adherence to LSI is poor (indicated by LDL-C > 140 mg/dL [3.6 mmol/L]), nutraceuticals/nutraceutical combination [36] and/or low-dose statin therapy and/or ezetimibe (especially in those with statin-related muscle symptoms or not willing to use statins) in combination with continued LSI might be warranted in order to reach the recommended LDL-C goals. This three-arm practical management is based on the real-world adherence to lifestyle changes, which is usually only 25–30%, and even worse in patients in primary prevention [37, 38]. Real-world adherence to lipid-lowering therapy is also suboptimal and poor adherence is associated with worse CVD outcomes [39].

Recent position papers produced by the International Lipid Expert Panel [40–42] and other Scientific Societies [43] have summarised the evidence relating to the cholesterol-lowering effects of nutraceuticals in general [41, 42] and in the specific situation of statin intolerance [40]. A wide range of nutraceuticals, alone or in combination therapy, have been shown to have favourable effects on lipid profiles. However, nutraceuticals are often evaluated in much smaller studies than phase 3 evaluations of conventional pharmaceuticals. Furthermore, variation in product composition between different preparations of similarly named nutraceuticals can lead to heterogeneity of response. Thus, the evidence basis for the safety and efficacy of nutraceuticals is generally less strong than conventional pharmaceuticals. The role of nutraceuticals should not be to replace conventional pharmaceuticals in settings where they have been shown to be effective, but to optimise treatment when lipid targets are not met. Lipid-lowering nutraceuticals include red yeast rice (with confirmed production quality/citrinin-free), bergamot, berberine, policosanol, artichoke, soluble fibre, and plant sterols and stanols [40, 44]. Although the LDL-C reductions elicited by these agents are generally modest compared with high-intensity statins and PCSK9-inhibitors, nevertheless, the ‘lower is better for longer’ approach to cardiovascular risk-reduction implies that a small reduction of LDL-C sustained over a long period of time would be expected to accrue a substantial benefit in terms of CVD risk reduction. This latter interpretation is supported by the observation that ezetimibe therapy, despite producing a relatively mild LDL-C lowering, also caused a significant reduction of CVD risk [13, 45]. Finally, when LDL-C remains > 115 mg/dl (3.0 mmol/L), despite LSI, and eventually nutraceuticals, statin and/or ezetimibe [41, 45] therapy should be considered. In the opinion of the authors, there is no need for a more intensive approach for low-risk patients, particularly as we do not have enough data on the effectiveness of such an approach in reducing CVOT. Furthermore, the potential for increased risk of statin-related adverse effects would clearly not be desirable and might influence the effectiveness of later therapy in this group of patients.

## Conclusions

*In summary*, even mild LDL-C elevations at a young age elevate CVD risk and are likely to have a greater unfavourable prognostic impact than similar elevations in older individuals, as has been recently confirmed [46]. Commonly used 10-year CVD risk scores are insufficiently sensitive to detect long-term CV risk in younger individuals. The recent recommendation by the ACC/AHA [21] to use lifetime risk scores in younger patients is pragmatic and sensible. The use of systems highlighting the long-term CV risk in younger individuals is desirable [47, 48], and practical strategies combining LSI, nutraceuticals, statins, and ezetimibe (as proposed in this commentary) should be used to modify elevated lifetime risk when it is detected. With respect to LDL-C, we know that ‘lower is better for longer’; therefore, we must allow this knowledge to be used to help those subjects (currently) at ‘low CVD risk’. Individuals who undertake approaches to reduce lipids at a lower age can benefit from a lower life-long exposure to LDL-C.

It is also important to emphasise that the unmet need for prevention and suitable treatment for these patients is likely to grow (with increasing numbers of such patients in the everyday clinical practice). Additionally, because of the paucity of data, well-designed studies are still necessary in order to answer the questions on the efficacy, safety, and cost-effectiveness of long-term interventions to reduce LDL-C in low-risk populations. Moreover, with the suggested approach, which is driven by changes in lifestyle, we emphasise the critical importance of non-pharmacological interventions, not only for those at high and very high risk, but equally (or, perhaps, more importantly) for those at low cardiovascular risk.

## Data Availability

Not applicable

## Abbreviations

CHD: Coronary heart disease
CVD: Cardiovascular disease
EAS: European Atherosclerosis Society
ESC: European Society of Cardiology
HDL-C: High-density lipoprotein-cholesterol
HMG CoA: 3-Hydroxy-3-methyl-glutaryl-CoA
LCD: Low-carbohydrate diet
LDL: Low-density lipoprotein
LDL-C: Low-density lipoprotein-cholesterol
LSI: Lifestyle interventions
MACE: Major adverse cardiovascular events
PAF: Population attributable fraction
PCSK9: Proprotein convertase subtilisin/kexin type 9
